# Supplementary material for: Genetic Diversity and Selective Signature in Dabieshan Cattle Revealed by Whole-Genome Resequencing
Source: Biology (Basel). 2022 Sep 8;11(9):1327. doi: 10.3390/biology11091327 (PMC9495734; doi:10.3390/biology11091327)
Supplement: Supplementary file 1 [file biology-11-01327-s001.zip › Figure S4.pdf]

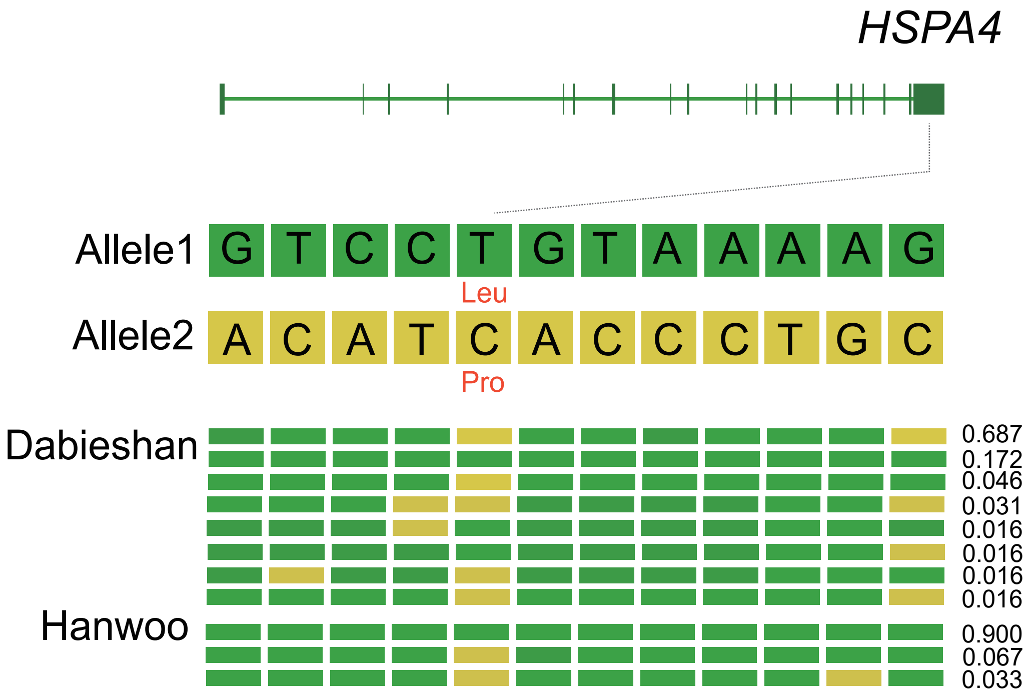

HSPA4
Allele1
G T C C T G T A A A A G
Leu
Allele2
A C A T C A C C C T G C
Pro
Dabieshan
Hanwoo
0.687
0.172
0.046
0.031
0.016
0.016
0.016
0.016
0.900
0.067
0.033
