# Supplementary figures and images for: Genetic Diversity and Selective Signature in Dabieshan Cattle Revealed by Whole-Genome Resequencing
Source: Biology (Basel). 2022 Sep 8;11(9):1327. doi: 10.3390/biology11091327 (PMC9495734; doi:10.3390/biology11091327)

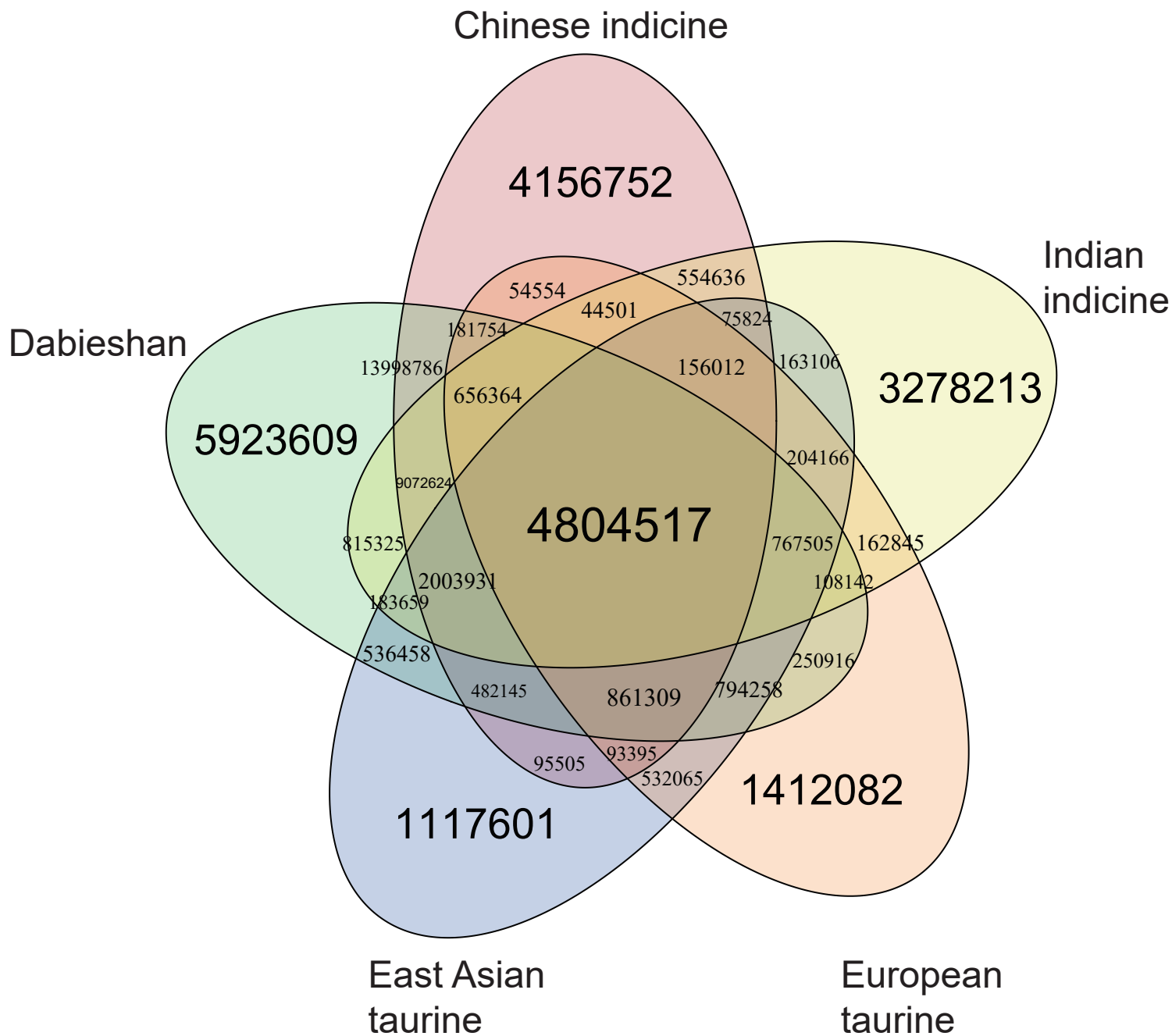

Supplement: Supplementary file 1 [file biology-11-01327-s001.zip › Figure S1.pdf]

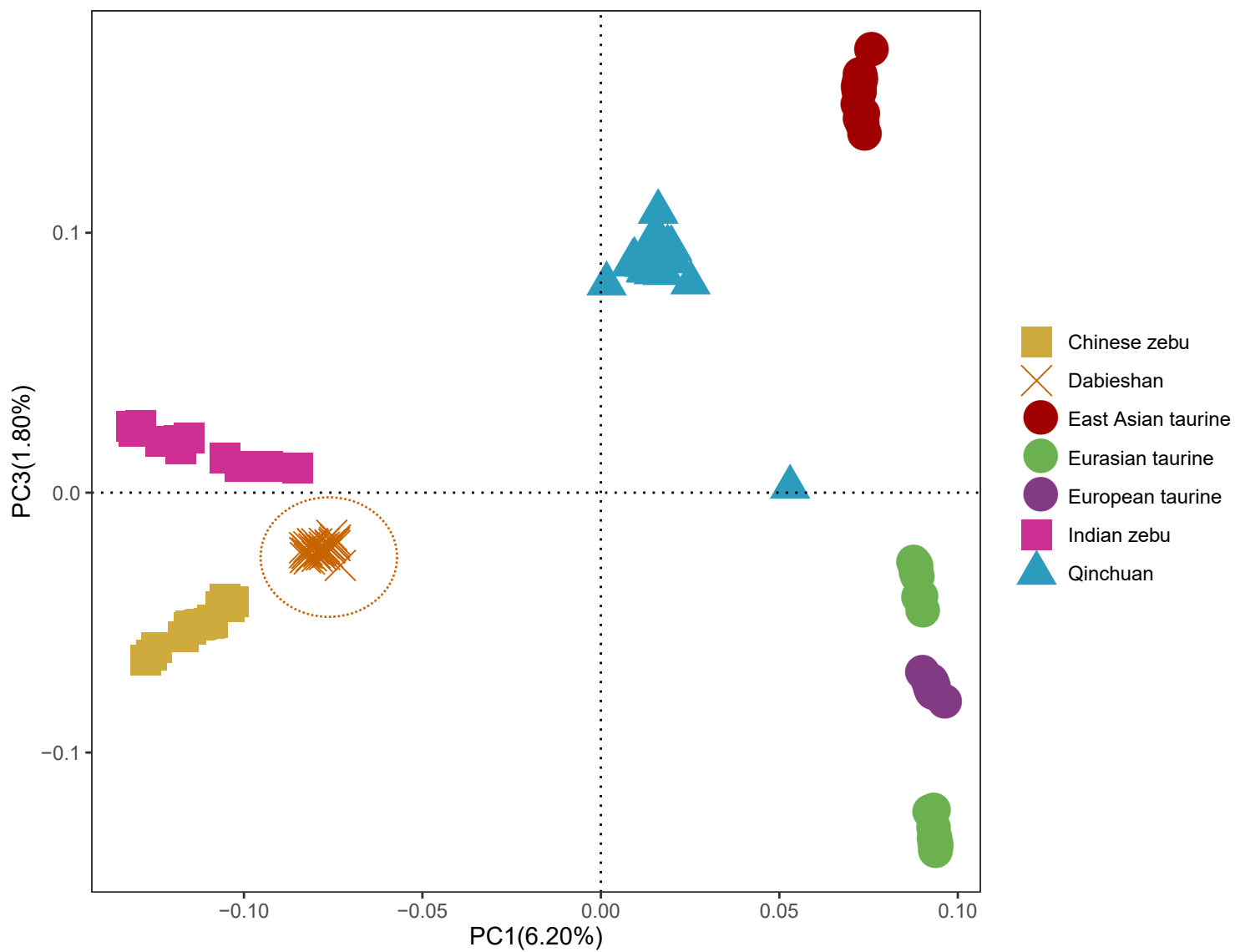

Supplement: Supplementary file 1 [file biology-11-01327-s001.zip › Figure S2.pdf]

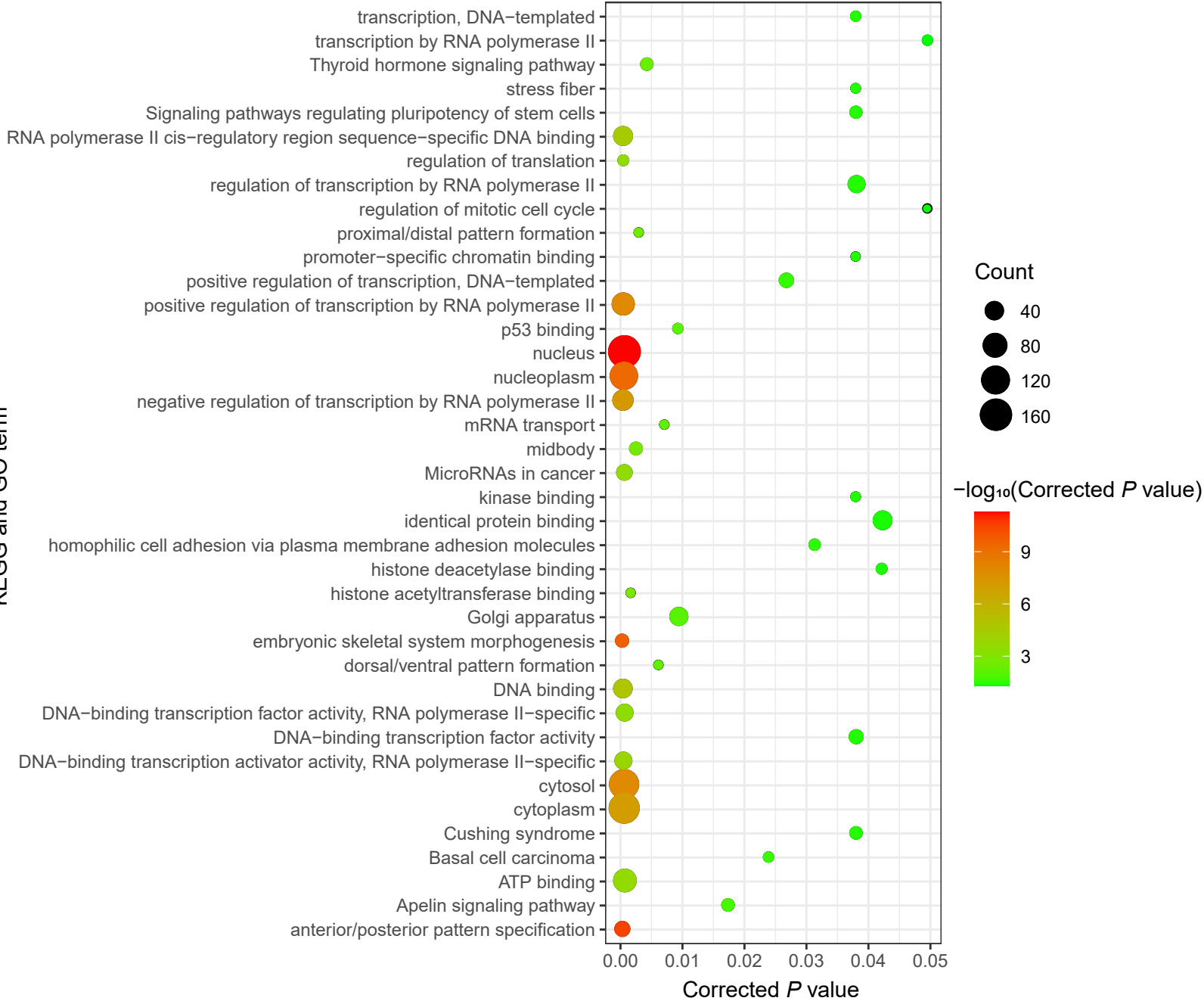

Supplement: Supplementary file 1 [file biology-11-01327-s001.zip › Figure S3.pdf]

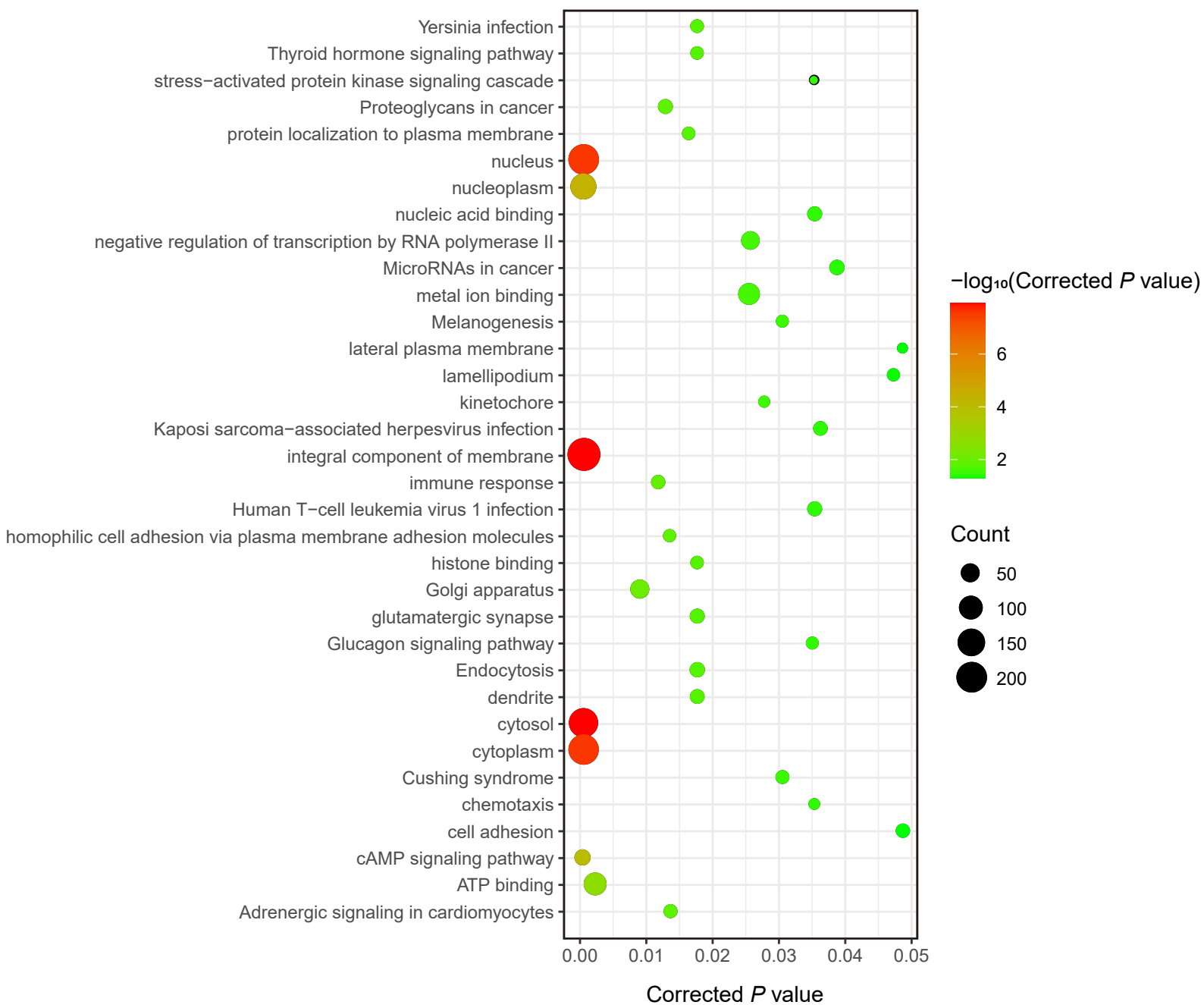

Supplement: Supplementary file 1 [file biology-11-01327-s001.zip › Figure S5.pdf]

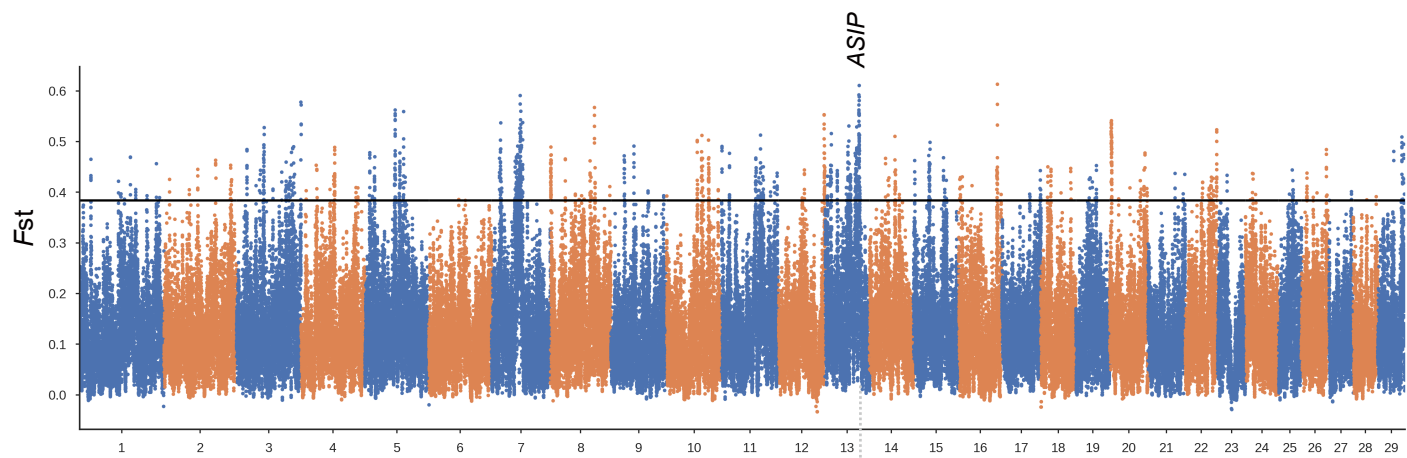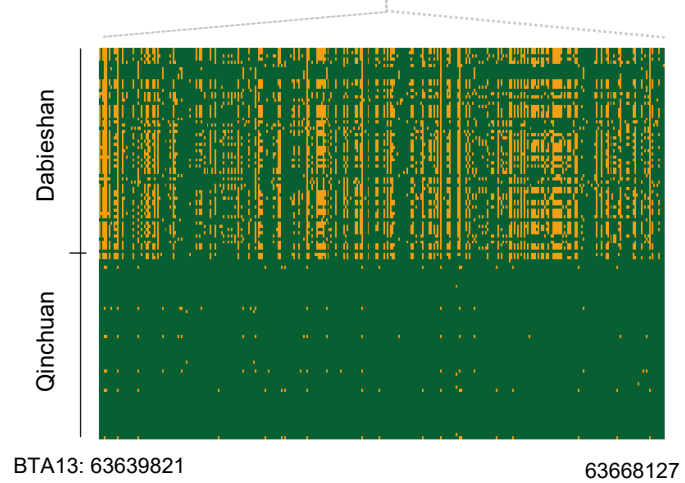

Supplement: Supplementary file 1 [file biology-11-01327-s001.zip › Figure S6.pdf]
